# Application of a structured perianal skin protection protocol incorporating individualized nutritional support for incontinence-associated dermatitis in active Crohn’s disease patients with diarrhea

**DOI:** 10.1097/MD.0000000000050344

**Published:** 2026-09-11

**Authors:** Yan Zeng, Mei Huang, Wenfang Hu, Yan Wu

**Affiliations:** a Department of Gastroenterology, The First People’s Hospital of Changde City, Changde City, Hunan Province, China; b Department of Burn, Plastic Surgery and Wound Repair, The First People’s Hospital of Changde City, Changde, Hunan Province, China.

**Keywords:** Crohn’s Disease, Incontinence-Associated Dermatitis, Individualized Nutrition, Propensity Score Matching, Structured Skin Care

## Abstract

This study evaluates the effect of a structured perianal skin protection protocol based on individualized nutritional intervention on diarrhea-related incontinence-associated dermatitis (IAD) in patients with active Crohn’s disease (CD). This retrospective cohort study included 200 active CD patients with diarrhea admitted from January 2023 to December 2025. Based on protocol implementation timing, patients were divided into a control group (January 2023–June 2024, n = 123) receiving routine perianal care, and an observation group (July 2024–December 2025, n = 77) receiving structured skin care plus individualized nutritional intervention. Propensity score matching yielded 65 matched pairs. IAD incidence, severity, perianal skin score, pain score, nutritional indicators, hospital stay, and costs were compared. Multivariate logistic regression analyzed IAD risk factors. After matching, the observation group showed significantly lower IAD incidence (30.8% vs 49.2%, *P* = .032) and better severity distribution (*P* = .031). Perineal skin condition score (5.1 ± 1.7 vs 8.3 ± 2.2) and pain NRS score (2.0 ± 1.0 vs 3.7 ± 1.4) were significantly lower in the observation group (*P* < .001). At discharge, albumin (38.5 ± 4.2 g/L vs 34.2 ± 4.1 g/L) and prealbumin (242.1 ± 50.3 mg/L vs 190.2 ± 46.5 mg/L) were significantly higher in the observation group (*P* < .001). Hospital stay was significantly shorter in the observation group (12.8 ± 4.1 days vs 16.5 ± 5.0 days, *P* < .001), with no significant difference in costs (*P* = .133). The observation protocol was an independent protective factor against IAD (OR = 0.38, 95%CI: 0.19–0.74, *P* = .004). The structured perianal skin protection protocol incorporating individualized nutritional support was associated with reduced IAD incidence and improved clinical outcomes in active CD patients with diarrhea.

## 1. Introduction

Crohn’s disease (CD) is a chronic nonspecific intestinal inflammatory disease of unknown etiology. As a major type of inflammatory bowel disease, it can affect the entire gastrointestinal tract, most commonly involving the terminal ileum and right colon.^[1]^ Characterized by recurrent episodes and a protracted course, its clinical manifestations include abdominal pain, diarrhea, weight loss, and nutritional disorders.^[2]^ Due to exacerbated intestinal inflammation during active disease, patients often present with intractable diarrhea, with bowel movements occurring several times or even more than ten times daily. The stool is typically watery or mushy (Type 6 or 7 on the Bristol Stool Form Scale).^[3]^ Studies indicate that approximately 70–80% of patients with active CD experience diarrhea of varying severity, significantly impacting their quality of life.^[4]^

Incontinence-Associated Dermatitis (IAD) refers to inflammatory skin changes resulting from prolonged or repeated exposure to urine or feces. Clinical manifestations include erythema, maceration, erosion, and even skin breakdown, often accompanied by pain or pruritus.^[5]^ For patients with active CD and diarrhea, the risk of IAD is significantly elevated.^[6]^ This is due to feces containing high levels of digestive enzymes (e.g., proteases, lipases) and bile salts, resulting in an alkaline pH that is more corrosive and irritating to the perianal skin. Coupled with frequent bowel movements that keep the skin persistently moist, the stratum corneum barrier function becomes impaired. Research shows that IAD incidence in hospitalized patients with diarrhea ranges from 30% to 50%, with potentially higher rates in those with active CD.^[7]^ IAD not only causes significant pain and discomfort, increasing the risk of secondary pressure injuries and fungal infections, but it may also lead patients to reduce food intake due to fear of defecation, thereby exacerbating malnutrition and creating a vicious cycle.^[8]^

Currently, clinical prevention and care for IAD primarily focus on skin cleansing and the application of protectants, such as using warm water for cleaning followed by applying zinc oxide ointment or petrolatum to form a physical barrier layer.^[9]^ However, for patients with active CD, conventional care protocols have certain limitations: on one hand, the timeliness and effectiveness of routine care are difficult to guarantee due to frequent diarrhea; on the other hand, improper cleaning methods (e.g., using regular wipes or vigorous wiping) may cause secondary skin damage. More importantly, routine care often overlooks the prevalent nutritional issues in CD patients. Due to intestinal absorption dysfunction, reduced intake, and a hypercatabolic state, patients with active CD frequently suffer from protein-energy malnutrition, manifested as hypoalbuminemia and weight loss. Studies have confirmed that low albumin levels are a significant risk factor for IAD development, as protein malnutrition leads to diminished skin repair capacity, making it difficult for damaged skin to heal.^[6,9]^ Therefore, superficial skin care alone can only provide a temporary barrier effect and cannot fundamentally address the issues of skin damage and repair.

In recent years, structured skin care protocols have gained increasing attention for application in incontinent patients. These protocols emphasize a risk assessment-based graded care strategy, including the use of skin-friendly cleansers with a pH close to that of the skin, employing a patting rather than wiping technique for cleaning, and the layered application of skin protectants or dressings according to skin condition, aiming to minimize skin irritation and damage.^[10,11]^ Concurrently, individualized nutritional intervention, as a crucial means of promoting tissue repair, has demonstrated positive effects in areas such as pressure ulcer care and wound healing.^[12]^ Integrating nutritional support with skin care to achieve a synergistic effect of “internal repair plus external protection” may represent an effective strategy to reduce the risk of IAD in patients with active CD. However, reports on the application of individualized nutritional intervention combined with structured skin care specifically in active CD patients with diarrhea remain scarce.

Therefore, this study employed a retrospective cohort design, utilizing propensity score matching to balance confounding factors, to investigate the application effect of a structured perianal skin protection protocol based on individualized nutritional intervention in patients with active CD accompanied by diarrhea. By comparing this protocol with routine care in terms of IAD incidence, severity, perianal skin condition, pain level, improvement in nutritional indicators, and hospitalization outcomes, this study aims to provide evidence-based support and clinical reference for perianal skin care in patients with active CD.

## 2. Materials and methods

### 2.1. Study subjects

This study was approved by the Ethics Committee of The First People’s Hospital of Changde City. This study employed a retrospective design. Based on predefined inclusion and exclusion criteria, patients with active Crohn’s disease accompanied by diarrhea, admitted to our hospital from January 2023 to December 2025, were selected as the study subjects.

**Inclusion criteria:** ① Diagnosed with Crohn’s disease according to the consensus on diagnosis and treatment of inflammatory bowel disease, and classified as active phase (CDAI > 150 or confirmed by endoscopy/imaging); ② Experienced frequent diarrhea during hospitalization (bowel movements ≥ 3 times/day, with stool classified as Type 6 or 7 on the Bristol Stool Form Scale); ③ Age ≥ 18 years; ④ Complete clinical medical records, including continuous skin care documentation.

**Exclusion criteria:** ① Presence of perianal skin breakdown, fungal infection, or pressure injury upon admission physical examination; ② Complicated with active anal fistula or perianal abscess requiring surgical intervention; ③ Presence of an enterostomy or urostomy; ④ Complicated with other severe skin diseases (e.g., psoriasis, bullous dermatoses) that might interfere with observation.

## 3. Grouping and propensity score matching

### 3.1. Study grouping

Patients were assigned to groups based on the implementation timeline of the perianal skin care protocol in our hospital.

**Control group:** Patients admitted from January 2023 to June 2024 who received routine perianal skin care (n = 123).

**Observation group:** Patients admitted from July 2024 to December 2025 who received the structured perianal skin protection protocol based on individualized nutritional intervention (n = 77).

### 3.2. Propensity score matching

To minimize selection bias inherent in retrospective studies and balance baseline differences between groups, propensity score matching (PSM) was performed at a 1:1 ratio. The grouping variable was set as the dependent variable, with age, BMI, admission CDAI score, daily bowel movement frequency, and admission serum albumin level entered as covariates. Propensity scores were calculated for each patient using logistic regression. Nearest neighbor matching was applied with a caliper width of 0.002, without replacement. Ultimately, 65 matched pairs were successfully formed, yielding 65 patients in both the control and observation groups, totaling 130 patients for post-matching analysis. Baseline characteristics were well-balanced between the 2 matched groups (*P* > .05). Propensity score matching was performed to reduce imbalance in measured baseline characteristics between groups. However, because patients were assigned according to the implementation period of the protocol, residual confounding related to temporal trends and changes in clinical practice cannot be completely excluded.

## 4. Study methods (nursing intervention assessment)

### 4.1. Nursing protocol for the control group

Patients in the control group received routine perianal skin care and basic dietary guidance as practiced in our department. The specific measures were as follows: Upon patient admission, the responsible nurse closely monitored vital signs and diarrhea status, addressing any abnormalities promptly. After each bowel movement, the nurse immediately cleaned the perianal skin gently using a soft towel moistened with warm water or baby wipes to remove feces, avoiding vigorous wiping to prevent mechanical injury. Following cleaning, the skin was gently patted dry with a dry towel and allowed to air-dry naturally. Subsequently, a barrier cream (e.g., zinc oxide ointment) was evenly applied to the skin around the anus and the gluteal cleft to form a physical barrier layer. During each care session, the nurse carefully inspected the perianal skin for signs of redness, breakdown, or maceration. For patients with intact skin, the focus was on maintaining local cleanliness and dryness; for those with existing skin damage, appropriate film dressings were applied for protection after cleaning, based on the condition. Additionally, when the patient’s condition permitted, repositioning was assisted every 2 hours, and soft pillows were placed under pressure points such as the sacrococcygeal area for relief. Furthermore, nurses provided routine dietary guidance to patients and their families, advising regular meals, thorough chewing, increased intake of dietary fiber, avoidance of excessively sweet, greasy, or spicy foods, and maintaining a daily fluid intake of 1.5–2 L.

### 4.2. Nursing protocol for the observation group

Patients in the observation group received the structured perianal skin protection protocol based on individualized nutritional intervention, in addition to the routine care provided to the control group. This protocol emphasized multidisciplinary collaboration, risk assessment, precise protection, and nutritional repair. The specific implementation steps were as follows:

#### 4.2.1. Establishment of a Multidisciplinary Structured Care Team

A dedicated care team was formed, comprising the head nurse, primary nurses, a dietician, and the attending physician. The head nurse, serving as the team leader, was responsible for organizing systematic training for team members. Training content covered the pathogenesis of incontinence-associated dermatitis (IAD), pressure injury assessment, structured skin care procedures, and nutritional risk screening and intervention. The training employed a combination of PowerPoint lectures, case analyses, and workshops, totaling 4 credit hours. Only personnel who passed the subsequent assessment were permitted to participate in the study. The attending physician was responsible for managing the underlying disease and adjusting antidiarrheal medication. The dietician guided the formulation of individualized nutritional plans. Primary nurses were responsible for implementing the protocol and collecting data.

#### 4.2.2. Implementation of Risk Assessment-Based Structured Skin Protection

Within 2 hours of admission, the primary nurse systematically assessed the patient’s risk level for developing IAD and pressure injuries using the internationally recognized Perineal Skin Condition Assessment Tool and the Braden Pressure Ulcer Risk Scale. Based on this assessment, a graded care strategy was formulated. For high-risk patients, a warning sign was placed at the foot of the bed to remind staff to increase the frequency of inspections and care.

Cleaning Strategy: Immediately after each bowel movement, the perianal skin was cleaned gently using a specialized skin cleanser with a pH close to the skin’s physiological weak acidity, applied with a disposable soft nonwoven fabric. A patting rather than wiping technique was employed to avoid friction on the skin. After cleaning, the skin was gently patted dry with a dry towel or allowed to air-dry naturally; vigorous rubbing was strictly prohibited.

Protection and Repair Strategy: Once the skin was dry, layered protection was implemented based on the assessment results.

Intact Skin: A layer of skin protectant (liquid barrier film) was evenly sprayed onto the perianal area and buttocks, forming a transparent, breathable protective layer to isolate the skin from fecal irritants.

Mild Erythema (Grade 1 IAD): After cleaning, stoma care powder was applied to absorb local exudate and keep the area dry, followed by spraying with a skin protectant film to secure it.

Broken Skin (Grade 2 IAD): The wound surface was cleaned with warm saline. Based on the exudate level, a hydrocolloid or foam dressing was applied. An outer layer of 3M transparent dressing was used for reinforcement and to extend the protected area. Dressings were routinely changed every 3–5 days, or immediately if they became curled, dislodged, or leaked. Additionally, for bedridden patients, highly absorbent and breathable disposable briefs were used, and repositioning was assisted every 2 hours to avoid prolonged localized pressure.

#### 4.2.3. Implementation of Individualized Nutritional Intervention

The core innovation of this protocol lies in integrating nutritional support as the internal driver for skin repair, alongside skin protection. Within 24 hours of admission, the primary nurse completed nutritional risk screening and requested a consultation with a dietitian.

Nutritional Assessment and Goal Setting: Individualized daily caloric and protein intake goals were collaboratively formulated based on the patient’s weight, BMI, serum albumin, prealbumin levels, and daily diarrhea frequency.

Nutritional Delivery Route: Enteral nutrition was the first choice whenever the patient’s condition permitted. Based on the patient’s intestinal tolerance, the dietician prescribed an individualized enteral nutrition regimen in addition to the regular diet.

For patients with frequent diarrhea and poor intestinal function: A short-peptide enteral nutrition formula was recommended to reduce the intestinal burden and facilitate absorption.

For patients with relatively stable intestinal function: A whole-protein enteral nutrition formula was recommended, supplemented with glutamine at a daily dose prescribed by the physician, to promote the repair of intestinal mucosal epithelial cells and damaged skin tissue.

Effect Monitoring: Nurses daily recorded the patient’s actual nutritional intake and changes in bowel movements. Nutritional indicators were regularly rechecked, and the nutritional plan was dynamically adjusted accordingly. This ensured that care measures and nutritional support worked in synergy, achieving a combined effect of “internal repair plus external protection.”

## 5. Data collection

Using the hospital’s electronic medical record system and nursing documentation sheets, two trained researchers independently extracted the following data:

### 5.1. General demographic and baseline clinical data

Patient demographic characteristics and clinical data upon admission were collected, including: age, sex, body mass index (BMI), and disease duration (years). The Crohn’s Disease Activity Index score within 24 hours of admission, daily bowel movement frequency, and stool consistency (Bristol Stool Form Scale type) were also recorded. Additionally, clinical characteristics potentially influencing IAD occurrence were collected, including admission serum albumin level, use of biologics, use of corticosteroids, presence of diabetes mellitus, and smoking history.

### 5.2. Data on incontinence-associated dermatitis occurrence and severity

Based on the daily perianal skin condition descriptions in the nursing documentation sheets, and according to the IAD classification system recommended by the 2015 International Expert Panel on Incontinence-Associated Dermatitis, the occurrence of IAD during hospitalization (yes/no) was recorded for both groups. For patients who developed IAD, the most severe grade was further documented (Grade 0: No IAD; Grade 1: Mild IAD, intact skin but with redness; Grade 2: Moderate to Severe IAD, redness with skin breakdown, blisters, or erosion).

### 5.3. Data on perianal skin condition and pain scores

The daily Perineal Skin Condition Assessment Tool scores recorded in the nursing documentation were extracted; this scale evaluates dimensions including skin erythema, rash, and skin defects. Concurrently, the highest daily perianal skin pain score reported by the patient (Numerical Rating Scale score, 0–10) was recorded, with either the mean or worst value during hospitalization used for statistical analysis.

### 5.4. Data on nutritional indicators

Serum nutritional indicators measured within 24 hours of admission and at the final reexamination before discharge were collected, including: albumin, prealbumin, and hemoglobin levels. All indicators were uniformly tested by the hospital’s clinical laboratory; specific values and testing times were recorded.

### 5.5. Data on hospitalization outcomes

The total length of hospital stay (days from admission to discharge) and total hospitalization costs (in ten thousand Yuan) were recorded for both groups. Hospitalization cost data were extracted from the hospital billing system, encompassing all diagnostic, treatment, nursing, medication, and consumable expenses.

## 6. Statistical analysis

Data analysis was performed using SPSS version 26.0 statistical software. For measurement data, variables following a normal distribution were expressed as mean ± standard deviation (x̄ ± s), and comparisons between groups were conducted using the independent samples t-test. Variables not following a normal distribution were expressed as median and interquartile range, and comparisons between groups were conducted using the Mann–Whitney U test. Categorical data were expressed as frequency and percentage (%), and comparisons between groups were conducted using the chi-square test or Fisher’s exact test. Propensity score matching was employed to balance baseline differences between groups, with the grouping variable as the dependent variable and age, BMI, admission CDAI score, daily bowel movement frequency, and admission serum albumin level as covariates. 1:1 nearest neighbor matching was performed with a caliper width set at 0.02. To further adjust for confounding factors, multivariate logistic regression analysis was used to identify influencing factors for IAD occurrence. Variables with *P* < .10 in the univariate analysis were included in the model, and odds ratios with 95% confidence intervals were calculated for each factor. A *P*-value < .05 was considered statistically significant.

## 7. Results

### 7.1. Comparison of baseline characteristics between the two groups after propensity score matching

After propensity score matching, a total of 65 patient pairs were obtained (65 cases in the control group and 65 cases in the observation group) (Table 1). There were no statistically significant differences between the 2 groups in terms of age, sex, BMI, disease duration, admission CDAI score, daily bowel movement frequency, admission albumin level, use of biologics and corticosteroids, presence of diabetes mellitus, and smoking history (*P* > .05). This indicates that propensity score matching effectively balanced confounding factors between the 2 groups, resulting in good comparability of baseline characteristics.

**Table 1:** Comparison of general and baseline clinical characteristics between the two groups after matching.3+

Indicator	Control Group (n = 65)	Observation Group (n = 65)	Statistic	*P*-value
Age (years, $\bar{x}\pm s$)	42.8 ± 14.2	40.5 ± 13.1	t = 0.967	.335
Sex (Male/Female, n)	40/25	35/30	χ2*χ*2 = 0.782	.377
BMI (kg/m^2^, $\bar{x}\pm s$)	21.5 ± 3.4	20.8 ± 3.2	t = 1.201	.232
Disease Duration [years, M(Q_1_, Q_3_)]	5.2 (2.0, 9.5)	4.5 (1.8, 8.5)	Z = 1.023	.306
Admission CDAI Score (points, $\bar{x}\pm s$)	258.7 ± 52.3	245.6 ± 49.8	t = 1.456	.148
Daily Bowel Movement Frequency (times/day, $\bar{x}\pm s$)	6.3 ± 2.4	5.8 ± 2.1	t = 1.267	.208
Admission Albumin Level (g/L, $\bar{x}\pm s$)	32.1 ± 4.5	33.5 ± 4.6	t = 1.734	.085
Use of Biologics [n (%)]	30 (46.2)	26 (40.0)	χ2*χ*2 = 0.502	.479
Use of Corticosteroids [n (%)]	33 (50.8)	29 (44.6)	χ2*χ*2 = 0.492	.483
Complicated with Diabetes Mellitus [n (%)]	8 (12.3)	5 (7.7)	χ2*χ*2 = 0.773	.379
Smoking History [n (%)]	18 (27.7)	14 (21.5)	χ2*χ*2 = 0.656	.418

### 7.2. Comparison of perianal skin outcomes between the two groups after matching

To further evaluate the protective effects of different nursing protocols on perianal skin, the incidence and severity of IAD were compared between the 2 groups (Table 2). The results showed that the IAD incidence in the observation group was 30.8%, significantly lower than that in the control group (49.2%), with a statistically significant difference (*P* = .032). Regarding IAD severity, the observation group predominantly presented with mild cases (80.0%), while the proportion of moderate-to-severe IAD in the control group reached 37.5%. The difference in severity distribution between the 2 groups was statistically significant (*P* = .031).

**Table 2:** Comparison of IAD incidence and severity between the two groups after matching [n (%)].

Group	n	IAD Occurrence	IAD Severity Classification	
			Grade 1 (Mild)	Grade 2 (Moderate to Severe)
Control Group	65	32 (49.2)	20 (62.5)	12 (37.5)
Observation Group	65	20 (30.8)	16 (80.0)	4 (20.0)
Statistic		χ2*χ*2 = 4.615	Z = -2.156	
*P*-value		0.032	0.031	

### 7.3. Comparison of perianal skin condition and pain levels between the two groups after matching

The perianal skin condition and pain levels were assessed in both groups following the intervention (Table 3). The results showed that the perineal skin condition score in the observation group was 5.1 ± 1.7 points, significantly lower than that in the control group (8.3 ± 2.2 points, *P* < .001). The perianal skin pain NRS score in the observation group was 2.0 ± 1.0 points, also significantly lower than that in the control group (3.7 ± 1.4 points, *P* < .001).

**Table 3:** Comparison of perianal skin condition scores and pain scores between the two groups after matching (points, $\bar{x}\pm \;s$).

Group	n	Perineal Skin Condition Score	Perianal Skin Pain NRS Score
Control Group	65	8.3 ± 2.2	3.7 ± 1.4
Observation Group	65	5.1 ± 1.7	2.0 ± 1.0
t-value		9.234	7.89
*P*-value		<0.001	<0.001

### 7.4. Comparison of nutritional status improvement between the two groups after matching

Nutritional indicators were compared between the 2 groups at admission and discharge (Table 4). The results showed no significant differences in albumin, prealbumin, or hemoglobin levels between the 2 groups at admission (*P* > .05). At discharge, the observation group exhibited significantly higher levels of albumin (38.5 ± 4.2 g/L vs 34.2 ± 4.1 g/L) and prealbumin (242.1 ± 50.3 mg/L vs 190.2 ± 46.5 mg/L) compared to the control group, with statistically significant differences (*P* < .001). No statistically significant difference was found in hemoglobin levels between the 2 groups at discharge (*P* = .12).

**Table 4:** Comparison of nutritional indicators before and after intervention between the two groups after matching ($\bar{x}\pm \;s$).

Indicator	Control Group (n = 65)	Observation Group (n = 65)	*t*-value	*P*-value
Albumin (g/L)				
At Admission	32.8 ± 4.3	33.1 ± 4.4	0.389	.698
At Discharge	34.2 ± 4.1	38.5 ± 4.2	5.876	<.001
Prealbumin (mg/L)				
At Admission	169.8 ± 44.2	172.3 ± 45.1	0.312	.756
At Discharge	190.2 ± 46.5	242.1 ± 50.3	6.123	<.001
Hemoglobin (g/L)				
At Admission	110.8 ± 18.2	111.5 ± 18.6	0.212	.832
At Discharge	114.3 ± 17.5	119.2 ± 18.1	1.567	.12

### 7.5. Comparison of hospital stay length and hospitalization costs between the two groups after matching

The length of hospital stay and hospitalization costs were analyzed for both groups (Table 5). The results showed that the mean hospital stay in the observation group was 12.8 ± 4.1 days, significantly shorter than that in the control group (16.5 ± 5.0 days), with a statistically significant difference (*P* < .001). Regarding hospitalization costs, the observation group had slightly higher costs compared to the control group (48,200 ± 13,200 Yuan vs 44,800 ± 12,000 Yuan), but the difference was not statistically significant (*P* = .133).

**Table 5:** Comparison of hospital stay length and hospitalization costs between the two groups after matching ($\bar{x}\pm \;s$).

Group	n	Hospital Stay (days)	Total Hospitalization Costs (ten thousand Yuan)
Control Group	65	16.5 ± 5.0	4.48 ± 1.20
Observation Group	65	12.8 ± 4.1	4.82 ± 1.32
t-value		4.567	1.512
*P*-value		<0.001	0.133

### 7.6. Logistic regression analysis of factors influencing IAD occurrence after matching

To further adjust for potential residual confounding factors after matching and clarify the independent effect of the nursing protocol on IAD occurrence, multivariate logistic regression analysis was performed with IAD occurrence as the dependent variable, including group assignment and variables with *P* < .10 in the univariate analysis (Table 6). The results showed that, after adjusting for age, CDAI score, daily bowel movement frequency, and admission albumin level, the observation group nursing protocol was an independent protective factor against IAD occurrence (OR = 0.38, 95% CI: 0.19–0.74, *P* = .004), indicating that patients in the observation group had a 62% lower risk of developing IAD compared to the control group. Additionally, a high CDAI score (OR = 1.76, 95% CI: 1.18–2.62, *P* = .005) and high daily bowel movement frequency (OR = 2.05, 95% CI: 1.32–3.18, *P* = .001) were independent risk factors for IAD occurrence. Admission albumin level showed a protective trend but did not reach statistical significance (OR = 0.71, 95% CI: 0.49–1.03, *P* = .068).

**Table 6:** Multivariate logistic regression analysis of factors influencing iad occurrence after matching.

Variable	Reference	B	SE	Wald *χ*2	*P*-value	OR	95% CI
Group (Observation Group)	Control Group	-0.968	0.342	8.012	.004	0.38	0.19 ~ 0.74
Age (years)	Continuous	0.018	0.018	1	.317	1.02	0.98 ~ 1.05
CDAI Score (per 10-point increase)	Continuous	0.565	0.203	7.745	.005	1.76	1.18 ~ 2.62
Daily Bowel Movement Frequency (times/day)	Continuous	0.718	0.223	10.365	.001	2.05	1.32 ~ 3.18
Admission Albumin Level (g/L)	Continuous	-0.345	0.189	3.332	.068	0.71	0.49 ~ 1.03

## 8. Discussion

The results of this study demonstrate that a structured perianal skin protection protocol based on individualized nutritional intervention can significantly reduce the incidence of incontinence-associated dermatitis, alleviate the degree of skin damage, improve perianal skin condition and pain perception, effectively enhance patients’ nutritional status, and shorten hospital stay length in patients with active Crohn’s disease, “with no statistically significant difference in hospitalization costs between groups. This finding provides preliminary observational evidence regarding a potential multidisciplinary approach for perianal skin management with active CD.

Regarding IAD incidence, the observation group exhibited an IAD incidence of 30.8%, significantly lower than the 49.2% in the control group, which is consistent with findings from relevant domestic and international studies.^[13,14]^ Several potential mechanisms may explain this outcome. First, the risk assessment-based graded care strategy enabled precise intervention, providing early warnings for high-risk patients and increasing the frequency of care, reflecting a prevention-oriented nursing philosophy.^[15]^ Second, optimization of the cleaning strategy was a critical component. The use of a specialized cleanser with a pH close to the skin’s physiological weak acidity, combined with a patting rather than wiping technique, effectively removed fecal residue while minimizing mechanical friction damage to the stratum corneum.^[16]^ Furthermore, the application of a layered protection strategy was essential. The use of skin protectants, stoma care powder, hydrocolloid dressings, and other products based on skin condition achieved comprehensive coverage ranging from prevention to treatment, providing a practical and actionable clinical nursing protocol.^[17]^

Regarding IAD severity, among patients in the observation group who developed IAD, the majority presented with mild cases, with moderate-to-severe cases accounting for only 20.0%. In contrast, the proportion of moderate-to-severe IAD in the control group reached 37.5%, with a significant difference in distribution between the 2 groups. This finding suggests that the observation group’s nursing protocol not only prevents IAD occurrence but also effectively controls its progression once dermatitis develops. Analysis of the underlying reasons may relate to the early identification and timely intervention employed in the observation group. Systematic daily assessment enabled early detection of mild IAD signs such as skin erythema, allowing prompt intervention to interrupt inflammatory progression.^[18]^ Furthermore, individualized nutritional support may have contributed to these outcomes; however, because nutritional intervention was delivered together with structured skin protection measures, the independent effect of nutritional intervention cannot be determined from this study. Protein serves as the fundamental building block for skin tissue repair; adequate nutritional support provides the material basis for damaged skin repair, accelerating the healing of mild dermatitis and preventing its progression to moderate-to-severe stages.^[19]^

In terms of perianal skin condition and pain scores, the observation group demonstrated significantly lower perineal skin condition scores and pain NRS scores compared to the control group. Pain is one of the most distressing symptoms for IAD patients, with persistent burning and stinging sensations not only affecting patient rest and sleep but also potentially leading patients to deliberately reduce food and fluid intake due to fear of defecation, thereby exacerbating malnutrition.^[20]^ The significantly reduced pain scores in the observation group may be attributed to multiple factors: the use of skin protectants reduced direct stimulation of exposed nerve endings by fecal matter; hydrocolloid dressings provided a moist wound healing environment, avoiding the pain exacerbation associated with traditional dry therapy; and improved nutrition promoted skin healing and reduced inflammatory responses.^[19,20]^

In terms of nutritional indicator improvement, at discharge, the observation group exhibited significantly higher levels of albumin and prealbumin compared to the control group, whereas no significant differences existed between the 2 groups at admission. This finding robustly validates the effectiveness of individualized nutritional intervention. Prealbumin, with a half-life of only 2 days, serves as a sensitive indicator reflecting recent nutritional improvement; the significantly elevated prealbumin levels in the observation group indicate that the individualized nutritional intervention produced effects within a short timeframe.^[21]^ For patients with frequent diarrhea and poor intestinal function, short-peptide enteral nutrition formulas can be directly absorbed without requiring digestion. For patients with relatively stable intestinal function, whole-protein enteral nutrition supplemented with glutamine was administered. Glutamine not only promotes intestinal mucosal repair but also directly participates in skin fibroblast proliferation and collagen synthesis, providing support for perianal skin repair.^[21]^ The improvement in nutritional status and skin protection created a synergistic effect, with both components complementing each other to collectively promote patient recovery.

Regarding hospitalization outcomes, the observation group demonstrated a significantly shorter hospital stay compared to the control group, with a mean reduction of 3.7 days. Although hospitalization costs were slightly higher in the observation group than in the control group, the difference was not statistically significant. This finding carries important health economic implications, as shortened hospital stays not only conserve medical resources but also reduce indirect economic burdens on patients.^[22]^ The slightly higher hospitalization costs in the observation group may be attributable to the use of novel nursing consumables, as well as expenses associated with dietician consultations and enteral nutrition formulas. However, these additional investments did not lead to a significant increase in total costs; instead, cost containment was achieved through shortened hospital stays. These findings indicate that the protocol was not associated with a statistically significant increase in hospitalization costs; however, formal cost-effectiveness evaluation was not performed.^[22]^

Multivariate logistic regression analysis revealed that, after adjusting for age, CDAI score, daily bowel movement frequency, and admission albumin level, the observation group nursing protocol remained an independent protective factor against IAD occurrence, with an odds ratio of 0.38. This indicates that patients in the observation group had a 62% lower risk of developing IAD compared to the control group. This finding further confirms that the effectiveness of the observation group nursing protocol is robust and unaffected by confounding factors. Additionally, the analysis results identified high CDAI score and high daily bowel movement frequency as independent risk factors for IAD occurrence, suggesting that clinical nursing should focus particular attention on patients with high CDAI scores and frequent diarrhea, positioning them as the core population for IAD prevention.^[13]^

## 9. Study limitations

This study has several limitations. First, as a retrospective study, data collection relied on the completeness and accuracy of medical records, which may introduce information bias. Although propensity score matching was employed to balance confounding factors, unknown or unmeasured confounding variables may still influence the study results. Second, the study sample was derived from a single center with a sample size of 130 cases, limiting representativeness. The generalizability of the conclusions requires further validation through multicenter studies with larger sample sizes. Third, due to the retrospective nature of the study, certain details within the observation group’s nursing protocol could not be fully standardized, potentially resulting in some operational variability. Fourth, because the structured protocol was introduced as a time-dependent clinical practice change, intervention exposure was completely correlated with the hospitalization period. Although propensity score matching adjusted for several measured confounders, it could not fully eliminate potential secular trends, including improvements in clinical management, nursing experience, or other concurrent practice changes over time. Therefore, the observed associations should be interpreted cautiously and cannot be considered as representing a purely causal effect of the intervention. Additionally, some outcome measurements, including perianal skin condition assessment and IAD classification, were derived from nursing documentation generated during routine clinical care. Because nurses involved in protocol implementation also recorded clinical outcomes, observer expectation bias cannot be excluded. Future studies should incorporate independent outcome assessors or blinded evaluation methods to improve objectivity. Finally, this study only observed short-term outcomes during hospitalization and did not follow up on patients’ long-term prognosis after discharge; the long-term benefits of individualized nutritional intervention and structured skin care warrant further investigation.^[21]^

## 10. Conclusion

In conclusion, this retrospective cohort study suggests that a structured perianal skin protection protocol incorporating individualized nutritional support may be associated with reduced incidence and severity of incontinence-associated dermatitis and improved short-term clinical outcomes among active Crohn’s disease patients with diarrhea. However, because the intervention was introduced as a time-dependent practice change and consisted of multiple integrated components, the independent effects of individual elements cannot be determined. Future multicenter prospective studies with standardized and blinded outcome assessment are needed to confirm these findings and clarify the mechanisms underlying potential benefit.

## Author contributions

**Conceptualization:** Yan Zeng, Mei Huang, Wenfang Hu, Yan Wu.

**Data curation:** Yan Zeng, Mei Huang, Wenfang Hu, Yan Wu.

**Formal analysis:** Yan Zeng, Mei Huang, Wenfang Hu, Yan Wu.

**Funding acquisition:** Yan Wu.

**Investigation:** Yan Wu.

**Writing – original draft:** Yan Wu.

**Writing – review & editing:** Yan Wu.
